# Obstacles to home-based dietary management for caregivers of children with citrin deficiency: a qualitative study

**DOI:** 10.1186/s13023-022-02437-z

**Published:** 2022-07-08

**Authors:** Shuxian Zhang, Yun Du, Lingli Cai, Meixue Chen, Yuanzong Song, Lilan He, Ni Gong, Qingran Lin

**Affiliations:** 1 School of Nursing, Jinan University, Guangzhou City, 510632 Guangdong Prov. China; 2 The First Affiliated Hospital of Jinan University, Guangzhou City, 510630 Guangdong Prov. China

**Keywords:** Obstacles, Dietary management, Caregivers, Children, Citrin deficiency

## Abstract

**Background:**

Dietary management is the most important and effective treatment for citrin deficiency, as well as a decisive factor in the clinical outcome of patients. However, the dietary management ability of caregivers of children with citrin deficiency is generally poor, especially in East Asia where carbohydrate-based diets are predominant. The aim of this study was to identify the difficulties that caregivers encounter in the process of home-based dietary management, and the reasons responsible for these challenges.

**Results:**

A total of 26 caregivers of children with citrin deficiency were recruited, including 24 mothers, one father, and one grandmother. Grounded theory was employed to identify three themes (covering 12 sub-themes) related to the dilemma of dietary management: dietary management that is difficult to implement; conflicts with traditional concepts; and the notion that children are only a part of family life. The first theme describes the objective difficulties that caregivers encounter in the process of dietary management; the second theme describes the underlying reasons responsible for the non-adherent behavior of caregivers; the third theme further reveals the self-compromise by caregivers in the face of multiple difficulties.

**Conclusions:**

This study reflects the adverse effects of multi-dimensional contradictions on the adherence of caregivers to dietary management. These findings reveal that the dietary management of citrin deficiency is not only a rational process, rather it is deeply embedded in family, social, and dietary traditions.

## Background

As a rare disease for which there is no specific drug, citrin deficiency has been reported worldwide. Reasonable dietary management is the most important and effective treatment currently available [1]. If patients with citrin deficiency cannot receive and adhere to a lifelong diet with high protein, high fat, and low carbohydrate intake, it may lead to a series of complications, such as cataracts, failure to thrive, dyslipidemia, liver failure, and hyperammonemic encephalopathy [1–3]. Therefore, following the correct dietary principles has great therapeutic importance for patients with citrin deficiency [1, 4, 5]. However, the dietary management ability of caregivers of children with citrin deficiency is generally poor, especially in East Asia where carbohydrate-based diets are predominant [6]. China, as the country with the largest number of cases [7], urgently needs to clarify the reasons behind this phenomenon.

Infancy and early childhood are critical periods for the formation of dietary preferences in children with citrin deficiency [8]. Therefore, parents of children with citrin deficiency—as the main managers and caregivers in the early stage of the disease—play a crucial role in their dietary management. Before the child develops dietary preferences, the home-based dietary management behavior of the caregiver almost directly determines the patient’s dietary habits. So, which factors hinder the home-based dietary management of caregivers?

Thus far, research on citrin deficiency has mainly focused on pathogenesis [9, 10], genetic diagnosis [11, 12], and dietary treatment strategies [13, 14]. There is no research study investigating the difficulties caregivers experience in dietary management, and the reasons underlying for these challenges. Studies show that the carbohydrate intake in patients with citrin deficiency is significantly lower than that noted in the healthy population [13–15]. However, the existing evidence is unable to determine the standard for carbohydrate intake in patients with citrin deficiency. Therefore, developing and maintaining special dietary preferences is particularly important for individuals with citrin deficiency.

Some scholars have proposed that the most effective dietary intervention for patients with citrin deficiency is to allow them to choose their favorite foods without restriction, rather than to limit their options [13]. However, the establishment and maintenance of dietary preferences are affected by several factors, such as individual sensory abilities, physiological and psychological characteristics, and food attributes, they are also profoundly affected by the social environment, including family circumstances [13]. Therefore, the establishment and maintenance of dietary preferences is highly dependent on the following two conditions. First, caregivers should assist children in establishing the correct diet by helping them choose the correct types of food when developing dietary preferences. Second, after the formation of dietary preferences by children, these preferences will not be easily changed.

The aim of this study was to identify the difficulties that caregivers encounter in the process of home-based dietary management, and the reasons responsible for these challenges.

## Methods

### Design

Grounded theory is an ideal research method for fields in which limited research has been conducted. This study adopted a constructivist grounded theory and used semi-structured interviews to investigate factors in family circumstances that are not conducive to dietary management in patients with citrin deficiency.

### Participants and sampling

We purposefully recruited 26 participants from a tertiary hospital located in Guangzhou, Guangdong Province, China. The hospital has the largest study cohort of patients with citrin deficiency in China. The inclusion criteria for caregivers were as follows: (1) responsible for the primary care of children with citrin deficiency; (2) no history of mental illness; (3) ability of verbal expression and basic reading skills; and (4) willingness to voluntarily participate in the study. Employed caregivers were excluded from the study.

At the initial stage of the study, we adopted purpose sampling for the selection of participants. A pediatric specialist assisted in selecting potential participants and asked them if they were willing to participate in this study. After generating relevant categories through data analysis, we used theoretical sampling for the selection of participants [16]. Data saturation determined the sample size; that is, data analysis did not generate new categories, the attributes and dimensions of the existing categories have been fully described, the relationships between the categories have been clarified, and the theory formed has sufficient explanatory power [17]. The demographics of the participants are provided in Table 1 (at the end of the document text file).

**Table 1:** Descriptive characteristics of caregivers and patients (n = 26, respectively)

Characteristics	${\bar{\text{x}}}$ ± SD/n (%)
Caregiver	
Sex	
Male	1 (3.85%)
Female	25 (96.15%)
Degree of kinship	
Mother	24 (92.30%)
Father	1 (3.85%)
Grandmother	1 (3.85%)
Having religious belief	
Yes (Buddhism)	1 (3.85%)
No	25 (96.15%)
Educational level	
Primary school	1 (3.85%)
Junior high school	5 (19.23%)
Senior middle school or vocational high school	5 (19.23%)
College degree or above	15 (57.69%)
Employment	
Yes	19 (73.08%)
No	7 (26.92%)
Place of residence	
East China region	2 (7.69%)
South China region	21 (80.77%)
Central China region	2 (7.69%)
Southwest region	1 (3.85%)
Patient	
Age, years	4.65±3.429
Sex	
Male	15 (57.69%)
Female	11 (42.31%)

### Data collection

Twenty-six semi-structured interviews were conducted between May 2021 and December 2021. All interviews were recorded and transcribed verbatim by the first author. The transcripts were provided to the interviewees to ensure accuracy. The duration of the interviews ranged 22–106 min (average: 50 min). Prior to conducting the formal interview, we explained in detail the main purpose and content of this research to the participants, and the researchers arranged the appropriate time and place for the interview according to the wishes of the participants. In this study, face-to-face and online interviews were performed, and all interviews were conducted by the first author. There wasn’t anyone else present besides the participants and the first author. Before data collection, the researchers received relevant guidance and training about the interview. With the help of the semi-structured interview guide, we initiated the interview by determining the recent situation of children with citrin deficiency. Gradually, we gained an in-depth understanding of the daily dietary management behaviors of caregivers and the relevant difficulties they encountered. The semi-structured interview guide is shown in Table 2.

**Table 2:** Semi-structured interview guide

Questions
When did your child start dietary management?
How has your family life changed since your child was diagnosed?
How do you usually manage the diet of your child?
How do you feel about your grasp of the contraindications for this disease?
What factors do you think have influenced your dietary management behavior?
What troubles you most about the dietary management of this disease?
What support and help would you like to receive while taking care of your child?

### Data analysis

The data analysis was conducted jointly by a male medical anthropologist, 3 female nursing postgraduates and 1 female clinical nurse. And the generated codes were discussed once a week during a data analysis meeting. In the initial stage of data analysis, we assessed all data line by line to identify ideas that could further guide data collection and analysis [16]. In the next stage, we discovered and formed the most salient taxa in the data by focused coding [16]. Subsequently, we used theoretical coding to clarify the relationships between the categories generated by the focused coding and to render these relationships concrete [16].

A constant comparative method was employed at each stage of the data analysis [16]. In addition, we recorded the new ideas and insights generated during the process of data analysis by memo writing to promote the formation of theories [16]. And all participants provide feedback on the findings.

### Ethical consideration

This study was approved by the medical ethics committee of the first affiliated hospital of Jinan university. Prior to the formal interview, the caregivers provided written or oral informed consent for their participation. All collected information was confidential, and the personal information of participants will never be disclosed to others without the consent of the participants. All authors have approved the final article.

## Results

**Fig. 1:**
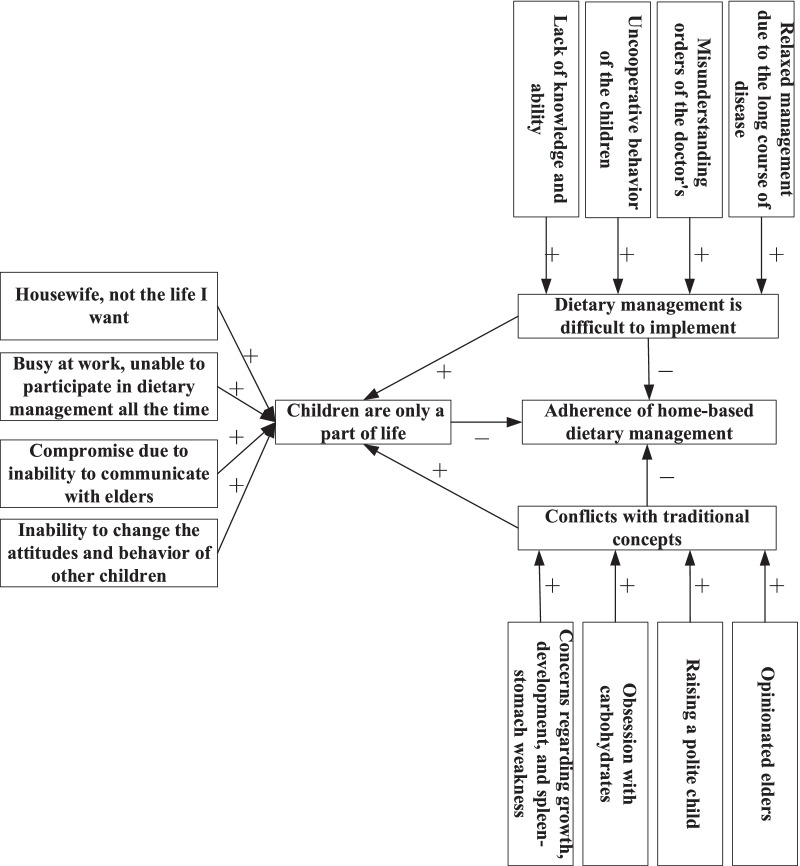
Obstacles to Home-based Dietary Management for Caregivers of Children with Citrin Deficiency

This study identified three themes related to the caregivers’ difficulties regarding home-based dietary management. The content and rationale of each theme are shown in Fig. 1.

### Dietary management is difficult to implement

This theme describes the objective difficulties that caregivers encountered in home-based dietary management. Lack of knowledge and ability, uncooperative behavior of children, misunderstanding of doctor’s orders, and continuity of the disease course were the main sources of difficulties for caregivers.

#### Lack of knowledge and ability

The culinary skills and abilities of the caregiver played an integral role in the home-based dietary management of children with citrin deficiency. Participants reported that their inability to cook increased the difficulty associated with dietary management.

It shouldn’t be difficult for a mother who is good at cooking, because I’m not good at cooking, so I think it’s a bit difficult. (P9).

However, dietary management remained a challenging task even for caregivers with superb culinary skills. After their children have been diagnosed with citrin deficiency, almost all respondents mentioned that they felt confused. Although they knew that citrin deficiency requires a high-protein, high-fat, and low-carbohydrate diet, they did not know how to determine the macronutrient composition ratio of foods. Furthermore, they did not know which foods could be used as staple foods or limited.

Inexperienced parents like us really don’t know how to judge…Many mothers don’t know anything at the beginning, they don’t know how to add [food], don’t know what can be eaten and what can’t be eaten, they are in a state of confusion…It’s really hard, sometimes it makes me want to cry. (P10).

#### Uncooperative behavior of the children

In the absence of relevant knowledge and ability, the children’s uncooperative behavior was undoubtedly aggravating. Respondents indicated that children’s non-cooperative behavior was also a common hindrance. They stated that the cooperation of children was required in both the adoption of a lactose-free formula and the introduction of solid food. Moreover, dietary management could become a daunting task following children’s refusal to eat the prepared food.

Unexpectedly, after adopting the lactose-free formula for less than one month, she began to hate it and didn’t want to drink it. She couldn’t stop crying when she was fed on lactose-free formula…It’s the same when she eats solid food, she is also very resistant to it now. (P24).

In addition, some of the participants reported that their children exhibited a strong preference for carbohydrates rather than developing typical dietary preferences, which was also a cause of frustration.

He likes rice very much. It makes me depressed, because sometimes he only eats rice, he really likes it. And he is willing to eat rice without anything else…He likes to eat rice, and he desperately wants to eat it. (P4).

Atypical dietary preferences of children affected the caregivers’ trust in special dietary principles and their interpretation of doctor’s orders.

#### Misunderstanding orders of the doctors

Participants reported that doctors advised them to give their children a small amount of carbohydrate-rich foods, such as rice and noodles. However, the caregivers had to follow the dietary preferences of their children and did not force them to eat foods they did not like. Consequently, the caregivers tended to selectively follow dietary advice that would be accepted by themselves, which led to deviations in dietary management and understanding.

I asked the doctor if my granddaughter still needed to drink lactose-free formula when we went to the follow-up in 2018. He said that my granddaughter didn’t need to drink lactose-free formula anymore, she could drink whatever she wanted, and eat whatever she wanted. (P5).

Then until he was two years old, the doctor told me that I could give him anything he wanted to eat…I thought it might be fine when my son turned two…I even didn’t know that he still couldn’t eat too much rice before this follow-up. (P8).

#### Relaxed management due to the long course of disease

Caregivers stated that, in addition to the doctor’s dietary recommendations, they also adjusted their dietary management behaviors based on the results of the follow-up examinations. If the laboratory test results were consistently within the normal range, the caregivers would appropriately relax the dietary management.

The follow-up found that his cholesterol was elevated…Because the results of his laboratory tests for the last two years were normal, we relaxed [dietary management] a little bit and gave anything he wanted to eat. (P16).

Moreover, long-term dietary management can easily tire the caregivers. Caregivers stated that with the growth of their children, they would gradually feel indifferent to dietary management. Moreover, occasionally they were willing to trust the choices and judgments of their children.

Doctor said [my son’s] cholesterol was elevated…It’s true, I have been strict [with the dietary management]in the past, but now I feel that I’m a little numb, anyway, he can judge by himself. (P19).

The above results summarize the influence of objective factors on the dietary management behavior of caregivers. However, in addition to objective factors, the strong conflict between tradition and modern medicine could also have a significant impact on the dietary management of caregivers.

### Conflicts with traditional concepts

This theme provides insight into the barriers that deep-rooted traditional thinking posed for caregivers, including concerns regarding the special dietary principles, obsession with traditional diets, emphasis on moral upbringing, and opinionated elders.

#### Concerns regarding growth, development, and spleen-stomach weakness

The dietary principles for the management of citrin deficiency differ considerably from traditional Chinese diets, and almost all respondents expressed concerns. If the dietary recommendations of the doctor did not match the dietary habits or life experiences of the caregivers, they would spontaneously develop a negative view of this approach. The participants reported that breastmilk was the best food in the world, and the recommendation to terminate breastfeeding raised concerns regarding the growth and development of their children.

There is a worry in my heart, because it is said that breastmilk is the best, but she stopped breastfeeding when she was three and a half months old and could only be fed on this artificially replaced milk powder. We worried about that her immunity would be a little worse than other children and her growth and development would also be slower than others…After all, artificial milk powder is not as good as natural food. (P3).

In addition, most respondents were biased in believing that a high-protein, high-fat, low-carbohydrate diet was unbalanced. They thought that adherence to this dietary principle would affect the growth and development of their children.

Her nutritional intake is not balanced. How could she grow up if she doesn’t get enough nutrients? I think she should keep the balance of food intake. (P1).

Many caregivers reported that they would seek help from a traditional Chinese doctor when their children showed signs of poor appetite. However, in most cases, they would be advised that the consumption of large amounts of meat can lead to spleen-stomach weakness in children. Recommendations from traditional Chinese doctors also posed another dilemma for caregivers.

I sought help from a traditional Chinese doctor, and he said my daughter got a spleen-stomach weakness…Now she needs to feed on high-protein food, but I think she can’t consume too much of it. From my perspective it’s because she eats too much high-protein food, so she gets the spleen-stomach weakness now. (P2).

#### Obsession with carbohydrates

In China, the traditional diet is primarily based on high-carbohydrate foods. Hence, it appears that all parents have a deep-rooted obsession with carbohydrates. The participants believed that high-carbohydrate foods were essential in their daily diet. Additionally, based on their opinion, only children who consumed rice were considered obedient, because caregivers firmly believed that children could be full only if they ate sufficient amounts of rice. Otherwise, they needed to spend time to convince their children to eat more.

But in my mind, “eating obediently” may mean that he can eat up all the rice I give to him, I think it will be easier to raise him up. But he just eats meats, so I always feel that he is not full. (P13).

It was under the influence of this traditional dietary concept that many caregivers stated they would subconsciously let their children eat more rice.

Sometimes I will give a little bit more rice to him because I’m afraid he won’t be full. Do you know that we Cantonese always think that we are not full if we don’t eat rice? (P19).

#### Raising a polite child

It is thought that the behavior of children is directly related to the style of parenting. Although caregivers knew that metabolic disorders could lead to special dietary preferences in children, they still felt that the picky eating behavior of their children in front of others was impolite. They did not want their children to develop a mentality of only considering their own preferences, so they would regulate their eating behavior.

He doesn’t like vegetables, but he likes to eat chicken and fried pork, he can finish the whole bowl of chicken by himself. I think it’s impolite. So, I always don’t allow him to eat too much. (P8).

Moreover, the respondents indicated that wasting food was also a bad habit. They did not want their children to reject the concepts that the society upheld because of the disease.

Sometimes I couldn’t help to force him to eat, such as a sandwich. Because I found that no matter how big the sandwich was, he always wanted to leave a part of it. I thought it was a bad habit, so I told him that he must finish it and couldn’t develop this habit. If he didn’t finish it, it would be a waste. (P13).

#### Opinionated elders

Participants reported that unreasonable intervention by elders hampered their dietary management efforts. Many participants reported that elders did not trust the dietary recommendations of doctors and thought it was ridiculous to restrict the intake of carbohydrates, insisting on their own principles.

The elders had never heard of this disease. They didn’t believe that the patients with citrin deficiency couldn’t eat too much rice…So they always tried to feed more rice to my daughter, and always inadvertently gave her a lot of lactose-containing or high-carbohydrate foods. (P15).

Moreover, many elders believed that their own life experience with regard to dietary management was more reliable than the doctor’s advice. This sense of distrust would lead them to disobey the doctor’s orders without hesitation.

Her grandma didn’t think it’s a problem [to eat a lot of rice], so she always tried to feed her some rice … Anyway, she thought that it was better to believe to herself rather than to the doctor. She felt that she had experienced a lot, and she thought that my daughter could eat rice without any restriction even though the doctor suggested us to restrict the intake of it. (P18).

Under the dual effects of objective and subjective factors, caregivers reported bad experiences associated with dietary management, such as fatigue and boredom. These negative effects further compromised their determination to insist on the dietary management of the children. Furthermore, they would naturally have the idea that children were only a part of life; hence, they could not let their children’s illness affect their lives and those of other family members.

### Children are only a part of life

This theme describes the conflicts that caregivers encountered when balancing dietary management of the children with family life. These included boredom with caregiving, being busy at work, compromise due to inability to explain the situation to elders, and the impact on other children’s needs of dietary management.

#### Housewife, not the life I want

Participants mentioned that, as mothers, they appeared to be inherently defined as the primary bearers of family care responsibilities. Therefore, some respondents stated that they were forced to quit their jobs to focus on the dietary management of their children. However, life with almost no social interaction and diminished financial resources induced feelings of great psychological pressure and helplessness. They were eager to escape from their families and pursue their own lives.

To be honest, I have taken care of them for so many years, I was also very tired, and I also wanted to have some social interactions. I can talk to someone else when I meet problems if I go to work. But at home I either contacted with my children, or contacted with the elders, I also had my own psychological pressure … When I asked my husband for money, do you think he didn’t complain? He would also ask me why I kept asking him for money. But if I go out to work, no matter how much money I make, it’s my own, right? (P18)

#### Busy at work, unable to participate in dietary management all the time

A proportion of the respondents reported that because of the financial burden of the family and the need for self-actualization, they would choose to work. Consequently, they had to juggle the dietary management with their own work responsibilities. They recognized that they could obtain the knowledge and experience regarding the dietary management for citrin deficiency through various channels, and they were also aware of the potential disobey behaviors with regard to the family circumstances. Nevertheless, because of the lack of sufficient time and energy, they were unable to resolve the existing problems.

Sometimes they also discussed [how to conduct the dietary management] in the WeChat group, but it was useless, because it was not me who cooked, it was cooked by my family members…After all, it was my mother, not me, who cooked lunch for my son. So, it was impossible to tell my son everything that he should pay attention and I was unable to participate in the dietary management all the time. (P4).

Furthermore, the respondents said that it was hard to convince elders to follow the correct dietary management principles. Only when they conducted the dietary management themselves, could they better control the carbohydrate intake of their children.

I think it was difficult [to obey the correct dietary principles] because sometimes the dietary management of my daughter was conducted by my mother. I probably wouldn’t let my daughter eat too much high-carbohydrate food if I would take care of her by myself. (P15).

#### Compromise due to inability to communicate with elders

Most respondents stated that they would choose to live with their elders because of the inheritance of traditional filial piety culture. However, they often experienced conflicts with elders due to differences in their concepts of dietary management. Nevertheless, to avoid further conflict, on most occasions, they would choose to compromise.

Her grandmother refused to accept my opinion, I had told her more than three times, and sometimes we even had some unpleasant conflicts, but she still rejected to accept it. What can I do? Quarrel with her every day? (P18).

Moreover, the participants indicated that the constant quarrelling failed to effectively reverse the acceptance of special dietary principles among elders and deteriorated the relationship between family members. Therefore, when caregivers found that communication did not solve the problem, they would choose to be silent.

I have told them about the cautions [of citrin deficiency], but I found they couldn’t accept them. And then they began to refute me, refuted me in various ways, and I even quarreled with them at the time…And I didn’t want to talk to them anymore when they rejected to accept my opinions. (P24).

#### Inability to change the attitudes and behavior of other children

As another important part of family life, the behavior and attitudes of other children could also have a significant impact on dietary management. Participants reported that other children in the family would become emotional because of the dietary management. They always thought that their parents were unfair, and they did not understand why their parents managed their diet in a different way.

My daughter thought that I gave too much meat to her little brother, so she couldn’t eat more meat as she wanted. She thought I was too partial to her little brother. (P19).

In addition, participants reported that they had emphasized the cautions regarding diet to other children. Nevertheless, in the absence of supervision, in some cases the children forgot these cautions.

Longan is too sweet, I usually didn’t give it to him, but sometimes his sister would give it to him when she took care of him…When I went to cook or did something else his sister would take care of him, and his sister liked to give him some snacks. (P11).

The three themes stemming from the results of the present study deeply reveal the difficulties that caregivers encounter in the process of home-based dietary management, reflecting the complexity of dietary management for citrin deficiency.

## Discussion

In this study, grounded theory was applied to investigate the difficulties that caregivers encountered in the process of home-based dietary management and the reasons responsible for these challenges. Although the participants of this paper are all from China, as the country with the largest number of cases, the problems we encountered are likely to be faced by families in other countries. They may be manifested in different forms, but the logic of dietary management of caregivers is likely to be the same. In China, the dietary management problems faced by families are manifested as: dietary management that is difficult to implement; conflicts with traditional concept; and the notion that children are only a part of family life. The first theme describes the objective difficulties that caregivers encountered in the process of dietary management. The second theme describes the underlying reasons responsible for the non-adherent behavior of caregivers. The third theme further reveals the caregiver’s self-compromise in the face of multiple difficulties. In summary, the contradiction between modern medicine and tradition, as well as that between dietary management and family life, constitute the main source of difficulties for caregivers.

The findings of this study suggest that the fragmentation of tradition by modern medicine is an important source of confusion and overwhelm for the caregivers. Because the dietary principle for citrin deficiency is almost completely opposite to the traditional Chinese dietary structure [13, 18]. Similar to other East Asian countries, carbohydrates occupy a very important place in the traditional Chinese diet [19, 20]. And it has shown that ingrained eating attitudes and habits is difficult to be changed [21, 22]. Therefore, almost all caregivers expressed their confusion and worry when the dietary advice provided by the doctor was in strong conflict with the traditional diet. And they would abandon medical advice in the long-term process of dietary management gradually. Furthermore, conflicting dietary recommendations between modern and traditional medicine also confused caregivers. Traditional treatment is another important factor affecting adherence to dietary management [23–25].

In addition, caregivers reported that a high-protein, high-fat, and low-carbohydrate diet was easily misunderstood by uninformed individuals. They did not want their children to move away from the accepted standard of “good children” as a consequence of the picky eating behavior. Owing to the dual influence of traditional culture and political strategy [26, 27], almost all parents attach great importance to the education and training of their children. Moreover, the inappropriate behavior of children is usually attributed to the parenting style employed by their parents [28, 29]. Therefore, understanding the contradiction between modern medicine and tradition is a prerequisite for adopting correct dietary management measures.

The conflict between dietary management and family life is another barrier for caregivers. There is no doubt that caregivers need to spend more time and effort with the children diagnosed with citrin deficiency. However, most caregivers could not effectively balance the relationship between diet management and work. For full-time caregivers, the lack of social life and self-actualization led to a poor caregiving experience, which could significantly affect their enthusiasm for dietary management. Difficulties in meeting both dietary management and their own needs, as well as emotional exhaustion, are common barriers that caregivers face [30]. However, the part-time care model hindered the timely prevention of potential unreasonable dietary management behaviors. Time constraint is another important factor affecting individual compliance to dietary management [21, 31, 32].

Home-based dietary management for citrin deficiency requires the cooperation of all family members. However, balancing the different opinions of elders and other children regarding dietary management is a difficult task. It seems very difficult for middle-aged and elderly individuals with deep traditional concepts to accept dietary principles incompatible with tradition [33]. And when communication failed to change the mindset of elders, caregivers compromised to maintain the peace and harmony of family life. Moreover, when dealing with the dissents of other children, caregivers were forced to be as fair as possible. Therefore, successful resolution of the conflict between dietary management and family life is necessary for caregivers to achieve the goals of dietary intervention.

We can find that the dietary management of citrin deficiency is not only a rational process, rather it is deeply embedded in family, social, and dietary traditions. Diet is a core cultural element of a society [34], and the degree of conformity between dietary habits and the principles of dietary management of citrin deficiency directly affects the family’s acceptance and implementation of the principles. Therefore, different countries may encounter different dietary management problems due to different dietary habits, but the behavioral logic responsible for these problems is common, that is, the conflict between the dietary management of citrin deficiency and the inherent living habits and concepts of caregivers.

Adopting a family-participatory health education method is essential to promote the efficiency of dietary management for citrin deficiency. It can improve cooperation among family members and reduce the burden of the primary caregiver. In addition, medical staff can regularly monitor the dietary intake in children through WeChat and remind the caregivers to visit the hospital with their children for follow-up examination as planned by the doctor.

## Limitations

This study was conducted in a specific cultural context in China, the applicability of the present results to other countries warrants further investigation. Although the results of this study have certain limitations in terms of cultural suitability, the behavioral logic in dietary management among different countries and regions is the same. In the future, we can conduct a multi-center study in conjunction with other countries to improve the cultural suitability of the findings. In addition, most caregivers who participated in this study were the mothers of the children. Although the primary bearer of family care responsibilities is the mother, the opinions and views of other family members are equally important. In future studies, we should also aim to increase the proportions of other family members.

## Conclusions

This study reflects the adverse effects of multi-dimensional contradictions on the adherence of caregivers to dietary management. These findings reveal that the dietary management of citrin deficiency is not only a rational process, rather it is deeply embedded in family, social, and dietary traditions. In addition, our findings can provide a basis for the formulation of individualized health education programs and promote the establishment and maintenance of the correct diet in patients with citrin deficiency, ultimately improve the efficiency of diet management.

## Data Availability

The data presented in this study are available on request from the corresponding author. The data are not publicly available due to [restrictions e.g., their containing information that could compromise the privacy of research participants].
